# Radiprodil, a NR2B negative allosteric modulator, from bench to bedside in infantile spasm syndrome

**DOI:** 10.1002/acn3.50998

**Published:** 2020-02-27

**Authors:** Stéphane Auvin, Blandine Dozières‐Puyravel, Andreja Avbersek, David Sciberras, Jo Collier, Karine Leclercq, Pavel Mares, Rafal M. Kaminski, Pierandrea Muglia

**Affiliations:** ^1^ Service de Neurologie Pédiatrique Hôpital Robert Debré Paris France; ^2^ Université de Paris INSERM U1141 F‐75019 Paris France; ^3^ UCB Pharma Braine‐L’Alleud Belgium; ^4^ Institute of Physiology The Czech Academy of Sciences Prague Czech Republic

## Abstract

**Objective:**

Infantile spasm syndrome (ISS) is an epileptic encephalopathy without established treatment after the failure to standard of care based on steroids and vigabatrin. Converging lines of evidence indicating a role of NR2B subunits of the N‐methyl‐D‐aspartate (NMDA) receptor on the onset of spams in ISS patients, prompted us to test radiprodil, a negative allosteric NR2B modulator in preclinical seizure models and in infants with ISS.

**Methods:**

Radiprodil has been tested in three models, including pentylenetetrazole‐induced seizures in rats across different postnatal (PN) ages. Three infants with ISS have been included in a phase 1b escalating repeated dose study.

**Results:**

Radiprodil showed the largest protective seizure effects in juvenile rats (maximum at PN12, corresponding to late infancy in humans). Three infants resistant to a combination of vigabatrin and prednisolone received individually titrated doses of radiprodil for up to 34 days. Radiprodil was safe and well tolerated in all three infants, and showed the expected pharmacokinetic profile. One infant became spasm‐free and two showed clinical improvement without reaching spasm‐freedom. After radiprodil withdrawal, the one infant continued to be spasm‐free, while the two others experienced seizure worsening requiring the use of the ketogenic diet and other antiepileptic drugs.

**Interpretation:**

Radiprodil showed prominent anti‐seizure effect in juvenile animals, consistent with the prevalent expression of NR2B subunit of the NMDA receptor at this age in both rodents and humans. The clinical testing, although preliminary, showed that radiprodil is associated with a good safety and pharmacokinetic profile, and with the potential to control epileptic spasms.

## Introduction

Infantile Spasms Syndrome (ISS) is an infantile epileptic encephalopathy. It is characterized by epileptic spasms occurring around 6 months of age and most frequently severe abnormal interictal electroencephalographic (EEG) activity, showing hypsarrhythmia or a similar pattern.^1^, ^2^

The etiology of ISS is diverse. The most frequently encountered etiologies are hypoxic‐ischemic encephalopathy, chromosomal abnormalities, monogenic conditions such as tuberous sclerosis complex, and brain malformations. There is substantial genetic heterogeneity in the ISS population with causative pathogenic variants involving more than 30 different genes,^3^ as recently illustrated in a study of 92 patients with ISS.^4^ However, in up to 40% of cases, the cause remains unidentified.^5^, ^6^, ^7^ Irrespectively of the genetic etiology, converging lines of evidence suggest a pathogenetic role of glutamate‐N‐methyl‐D‐aspartate (NMDA) transmission in ISS.^8^, ^9^

The incidence of ISS is 0.16–0.42 per 1000 live births.^10^ The prevalence is estimated to be 1–9/100,000, fulfilling the criteria for an orphan disease. Nevertheless, ISS is the single largest epilepsy syndrome in the infantile period.^11^ Onset of spasms is often associated with developmental arrest or regression. The primary goal of treatment is to stop the spasms early in the course of the disease in order to minimize the developmental delay.^12^, ^13^, ^14^ Up to 20% of patients fail to respond to current standard of care (SoC) with hormonal therapy, vigabatrin, or their combination.^14^, ^15^

The SoC treatments with proven efficacy include hormonal treatments (ACTH, tetracosactide, or prednisolone) and vigabatrin. Vigabatrin was the first drug approved for the treatment of ISS by the FDA. Although ACTH has been used in infantile spasms since the first description of its efficacy in 1958, approval by the FDA was obtained in 2010.^16^

Infants who fail to respond to SoC represent the drug‐resistant ISS population for which no established treatment is available. In those infants there is a clear unmet need for an efficacious treatment that will halt spams and related EEG abnormalities. Furthermore, such an efficacious treatment may eventually provide additional benefits to the entire ISS population as a safer alternative to the current standard of care.

## The NR2B subunit of NMDA receptor is a relevant target for early onset epilepsy: rationale for potential therapeutic use of radiprodil

N‐methyl‐D‐aspartate is one of the main excitatory glutamate receptors mediating a number of CNS functions including normal brain development and activity‐dependent synaptic plasticity.^17^ NMDA receptor dysfunction is also implicated in a range of neuropsychiatric disorders including epilepsy.^8^, ^9^ NMDA receptor antagonists have been shown to have antiepileptic effects including anecdotal reports of memantine use in epileptic encephalopathies.^18^ Converging lines of evidence suggest the NMDA receptor is involved in the disrupted electrical activity and generation of seizures observed in ISS. Activation of NMDA induces spasm‐like seizures in rat pups,^17^, ^19^ and systemic administration of NMDA is used as an ISS animal model characterized by interictal large amplitude asynchronous EEG abnormalities.^20^ During the first year of life in human, and during the corresponding age in rat pups, the composition of the NMDA receptor is prevalently constituted by the NR2B subunit. The increased neural excitability during early development, coinciding with the typical onset time of ISS, may be at least partially mediated by an increased expression of NR2B‐containing NMDA receptors.^17^, ^21^ Indeed, NR2B‐selective compounds, such as ifenprodil, showed an age‐dependent anticonvulsant effect in rodents, which appears consistent with the increased expression of NR2B containing receptors early in the development.^22^, ^23^ More recently, de novo gain of function mutations of the GRIN2B gene, coding for the NR2B subunit, have been reported as a rare cause of ISS.^24^, ^25^ It could be then hypothesized that the NR2B subunit is a therapeutic target for ISS, and that compounds inhibiting its activity have the potential to suppress spasms.

Radiprodil is an orally active, negative allosteric modulator of NR2B receptors.^26^ Interestingly, in vitro electrophysiological studies indicate that radiprodil fully retains its pharmacological profile in human NMDA receptors expressing the aforementioned NR2B gain of function mutations associated with ISS, while other nonselective NMDA receptor antagonists lose their potency.^26^ Consequently, radiprodil could be a candidate drug with the potential for controlling spasms. Radiprodil was initially developed as a treatment for neuropathic pain. The clinical experience with radiprodil was obtained in 460 adult individuals, including healthy volunteers in the phase 1 program and 332 patients with neuropathic pain. Development in neuropathic pain was terminated due to lack of efficacy, however these studies established a well‐tolerated exposure range in adults which supported the dose selection for the ISS study. To enable pediatric development, a juvenile toxicology study in rat pups was conducted, and the pediatric oral formulation was developed. Bioequivalence of the pediatric formulation was demonstrated (NCT02647697)^27^ and a microsampling technique for facilitating PK sampling in infant was validated.^27^

Here, we report the results of the in vivo preclinical studies in rodents, and the observed effects of radiprodil in three infants with ISS exposed in an open‐label phase 1b study with three individually titrated escalating doses of radiprodil administered for up to 34 days.

## Radiprodil: Preclinical Data

**Figure 1:**
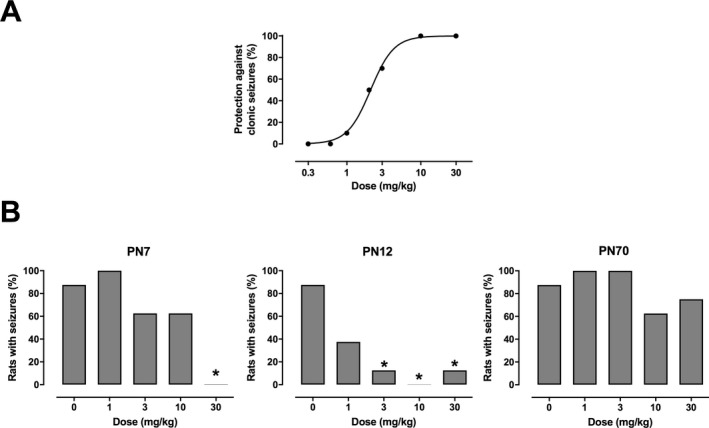
Anticonvulsant effects of radiprodil in rodent seizure models. (A) Dose‐dependent protection against generalized clonic convulsions in the mouse audiogenic seizure model. (B) Age‐dependent protection against generalized tonic seizures in the rat pentyleneterazol (PTZ) model at three different postnatal (PN) periods.

The anticonvulsant effects of radiprodil were assessed in two standard models of generalized and focal seizures in adult mice. In the audiogenic seizure model, radiprodil displayed very potent, dose‐dependent protective activity against generalized clonic convulsions, which are the key efficacy endpoint in this model. The obtained 50% effective dose (ED50) value was equal to 2.1 mg/kg (Fig. 1A). In contrast, radiprodil (up to 30 mg/kg) did not display any significant activity against focal seizures induced by electrical stimulation with 6 Hz current at 44 mA (data not shown).

The anticonvulsant activity of radiprodil was also tested in male Wistar rats at different PN ages, that is, PN7, PN12, and PN70, using pentylenetetrazole (PTZ) as a convulsant which typically induces generalized tonic‐clonic seizures in all age groups.^28^ Radiprodil showed dose‐dependent protective effects against the tonic phase of generalized seizures only in PN7 and PN12, while it did not have any significant anticonvulsant activity in PN70 rats injected with PTZ (Fig. 1B). The highest potency of radiprodil was observed in PN12 rats, approximately corresponding to infantile‐early childhood in humans. At 3 mg/kg radiprodil showed a statistically significant protective effect against the tonic phase of PTZ‐induced convulsions, while at 10 mg/kg it completely abolished tonic seizures in PN12 rats.

## Radiprodil: Clinical Results of Phase 1b Testing in ISS Patients

The clinical study was a phase 1b, open label, ascending dose adaptively designed study (NCT02829827).

The first two participants were enrolled as sentinel subjects before the inclusion of any additional infants. The aim was to include 10 participants in the first part of the study until an interim analysis, but the study was stopped earlier because of the challenge in recruiting patients meeting all inclusion and exclusion criteria.

1. The inclusion criteria were as follows:
  ‐ infant between 2 and 14 months of age with a diagnosis of ISS.
  ‐ The diagnosis requirement included both confirmation of the clinical features of ISS and evidence of hypsarrhythmia or other disordered interictal patterns consistent with the diagnosis of ISS on 4‐h video‐EEG.
  ‐ failure to respond to SoC defined as continuing spasms despite treatment with vigabatrin and one of the hormonal treatments (including ACTH, tetracosactide, and prednisolone), given in combination or sequentially for a minimum of 14 days.
2. The exclusion criteria were as follows:
  ‐ a diagnosis of ISS more than 6 months before screening
  ‐ a structural brain abnormality that likely required surgery during the study
  ‐ a clinically significant abnormality on electrocardiogram
  ‐ any previous or ongoing medical condition that could have significantly interfered with the safety, tolerability and/or absorption, metabolism, and excretion of the study drug.

### Study drug

Radiprodil was supplied as granules in bottles for oral suspension. The granules were reconstituted as a suspension prepared extemporaneously by adding 40 mL of diluent. Three dose levels of radiprodil were administered b.i.d.: 0.04 mg/kg (Low), 0.10 mg/kg (Medium), and 0.21 mg/kg (High).

#### Rationale for dose selection

The selection of the first lower dose and the upper dose limit was determined using available data from previous clinical experience with radiprodil and based on a number of considerations on the known NR2B pharmacology. First, safety considerations were based on the observation that 30 mg single or multiple dose were considered safe and well tolerated in adults also after long‐term (14‐week) treatment. The drug exposure associated with 30 mg was expected to achieve a maximum of 80% NR2B occupancy, that is below the uneventful drug concentration achieved in the juvenile toxicology dose (projected to achieve ~95% NR2B occupancy). Second, the lower drug concentration associated with anti‐seizure efficacy in the preclinical rat pup PTZ model was observed with 60% NR2B occupancy, and the expected NR2B receptor occupancy associated with a dose of felbamate, that has affinity for NR2B, and is effective in ISS is around 30%.^29^ Third, a physiologically based pharmacokinetic (PBPK) model, derived from the data in two clinical pharmacology studies mentioned above and its adaptation for pediatric simulations was used to establish doses that would be expected to produce the desired concentrations in infants. Simcyp Population‐Based Simulator (v. 15.1) was used for all simulations. The starting dose of 0.04 mg/kg was selected as being safe and expected to produce NR2B occupancy of approximately 20%, 28‐fold lower than the no‐observed‐adverse‐effect level (NOAEL) exposure in the juvenile rat toxicology study (100 mg/kg/day), and four times lower than the 30 mg of radiprodil in adult subjects considered safe and well tolerated. The high dose was capped at drug concentrations expected to achieve 60% NR2B occupancy which was judged to be the limit of a positive benefit/risk ratio.

### Study design

The study was approved by the ethic review board and by the French competent authority (Agence Nationale de Sécurité du Médicament et des Produits de Santé). After obtaining informed consent from the parents or caregivers, each participant underwent a baseline evaluation including a 4‐h video‐EEG.

Study participants were eligible to enter the study if they continued to have spasms despite treatment with vigabatrin and one of the hormonal treatments, given in combination or sequentially for a minimum of 14 days. Participants taking hormonal treatments at the time of enrollment had their treatment discontinued in a tapered fashion. Participants taking vigabatrin continued on a stable dose. It was recommended that any other antiepileptic drugs for non‐ISS seizures should have been given on a stable regimen during the radiprodil treatment period.

**Figure 2:**
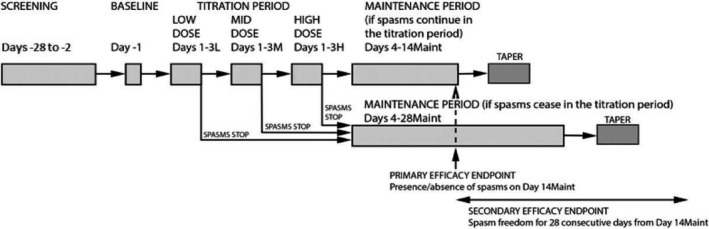
Design of the phase Ib study.

The radiprodil treatment period consisted of the titration, maintenance, and tapering periods. Following the baseline evaluation, each study participant entered an individualized dose titration schedule. The titration included three dose levels of radiprodil: 0.04 mg/kg (low), 0.10 mg/kg (medium), and 0.21 mg/kg (high dose) given twice daily (bid) for 3 days (Fig. 2).

The decision on whether to increase the dose was made in the morning on the day following the third day of a specific dose level, based on the effect on spasms (clinical and 1‐h video‐EEG on the previous day) and if there were any safety/tolerability issues. The decisions were taken during a Safety Review Meeting between the study team and the investigators. If no spasms occurred in the 24‐h period from 7 am Day 3 to 7 am Day 4 b.i.d. dosing was continued at the current dose level as the maintenance dose. For study participants whose doses were titrated to the high dose, if the spasms were still present on Day 3 of the high dose, the high dose was maintained and spams were reassessed on Day 14. If spasms were still present on Day 14, radiprodil was tapered and then discontinued. For study participants who were spasm‐free on Day 14, the radiprodil dosing was continued until Day 28; following Day 28, it was tapered and discontinued.

## Clinical Results

**Figure 3:**
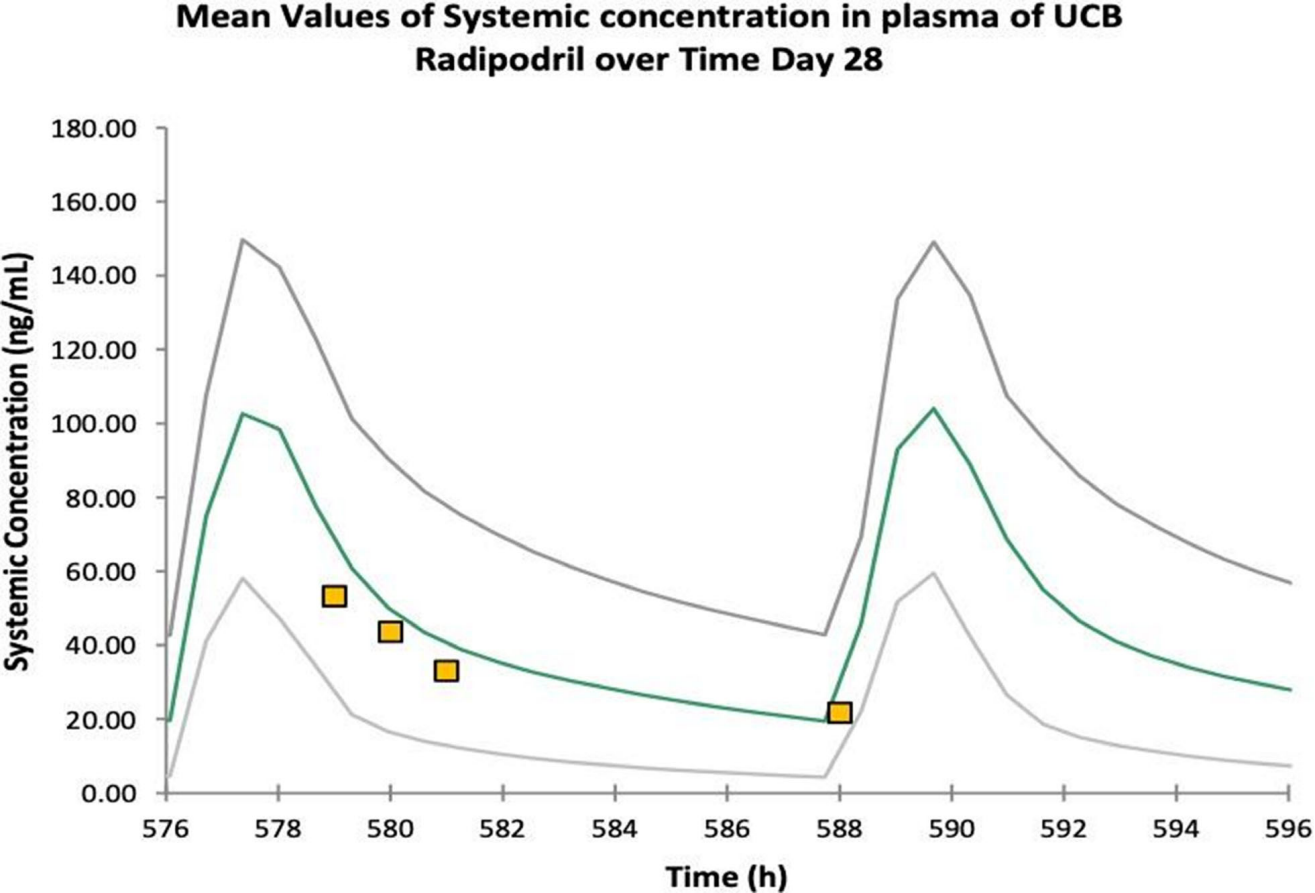
Predicted and observed Radiprodil plasma concentrations. The curves represent the modeling‐based projected plasma concentrations (green curve is mean ± 95% confidence limits) for the mid dose selected for the clinical trial after 28 days of dosing. The individual data points are the observed concentrations for this dose in the first infant in the trial.

Three participants were included and treated with radiprodil, entered into the maintenance period, and completed the treatment period. All three participants received all scheduled doses, plasma exposure both in terms of C_max_ and AUC_(0‐_ *_t_*_)_ increased with dose in a linear fashion (Table 1) as expected based on the PKPB modeling (Fig. 3).

‐ Study participant #1 was male, 11 months old at screening. He was 10 months old at diagnosis of ISS due to a malformation of cortical development (right insular polymicrogyria).
‐ Study participant #2 was female, 5 months old at screening. She was 4 months old at diagnosis of ISS of unknown underlying etiology. She had a clinically significantly neurological history (abnormal periventricular white matter on MRI, pyramidal and extrapyramidal disorder, and developmental delay).
‐ Study participant #3 was male, 7 months old at screening. He was 5 months old at diagnosis of ISS and had initially an unknown underlying etiology but was later diagnosed with a large malformation of cortical development.

**Table 1:** Pharmacokinetic parameters.

Study participant visit	Dose (mg/kg)	AUC _(0‐_ *_t_*_)_ (ng/mL·h)	*C*_max_ (ng/mL)	*C*_min_ (ng/mL)	*t*_max_ (h) (median)	*t*_½_ (h)
Patient #1						
Day 2L	0.04	214	47.1	7.5	1.25	6.7
Day 7Maint	0.1	535	113.8	18.9	1.69	6.8
Day 14Maint	0.1	535	113.7	18.9	1.69	6.7
Patient #2						
Day 2L	0.04	185	44.3	5.28	1.15	5.3
Day 8Maint	0.21	462	107.2	13.9	1.4	5.3
Day 14Maint	0.21	969	208.4	30.0	1.85	5.3
Patient #3						
Day 2L	0.04	198	45.8	6.3	1.2	5.9
Day 7Maint	0.21	495	110.3	16.0	1.5	5.9
Day 14Maint	0.21	1036	207.8	34.7	1.9	5.9

PK parameters were derived from a pop‐PK model.

*T*_max_ was the median.

L, radiprodil low dose that is, 0.04 mg/kg; Maint, maintenance; PK, pharmacokinetic; SS, Safety Set.

All included patients had abnormal development prior to/after the onset of ISS, had a normal metabolic screening test, and took a wide range of prior anticonvulsants (Table 2). No clinically significant changes from baseline in vital signs or physical measurements were observed during the overall treatment period with radiprodil. The duration of exposure to radiprodil ranged from 26 to 34 days.

**Table 2:** Individual efficacy responses.

	Included patients		
	Patient #1	Patient #2	Patient #3
Maintenance dose	Medium	High	High
Underlying cause of ISS	MCD	Unknown	Unknown MCD found after the study
Previous AED	VPA, LTG, LVT		
AED at inclusion	VPA 26 mg/kg/d, LTG 1.7 mg/kg/d, VGB 130 mg/kg/d, Prednisolone 10 mg/d	VGB 130 mg/kg/d prednisolone 10 mg/d	VGB 146 mg/kg/d prednisolone 20 mg/d
Clinical responder	Y	N	N
Electroclinical responder	Y	N	N
Time to cessation of spasms for clinical responders (days)	6	NA	NA
Interictal EEG at Baseline	Slow waves	Hypsarrhythmia	Fragmented
	Right fast rhythm	Post Spikes	Hypsarrhythmia
			L Temp Spikes
Interictal EEG at the end of the maintenance period	No change	No change	No change
Extended clinical responder	Y	NA	NA
Extended electroclinical responder	Y	NA	NA
Clinical relapse	N	NA	NA
Clinical worsening with radiprodil withdraw	NA	Y	Y
Last follow‐up			
Time after inclusion	M11	M10	M10
Clinical follow‐up	Spasm‐free	Spasms‐free	Spasms‐free
AEDs	VPA, LTG	FLB, KD	VPA, LTG, FLB, KD Surgery

AED, antiepileptic drug; FLB, felbamate; KD, ketogenic diet; LTG, lamotrigine; LVT, levetiracetam; MCD, Malformation of cortical development; N, No; NA, not applicable; VGB, vigabatrin; VPA, valproate; Y, Yes.

**Figure 4:**
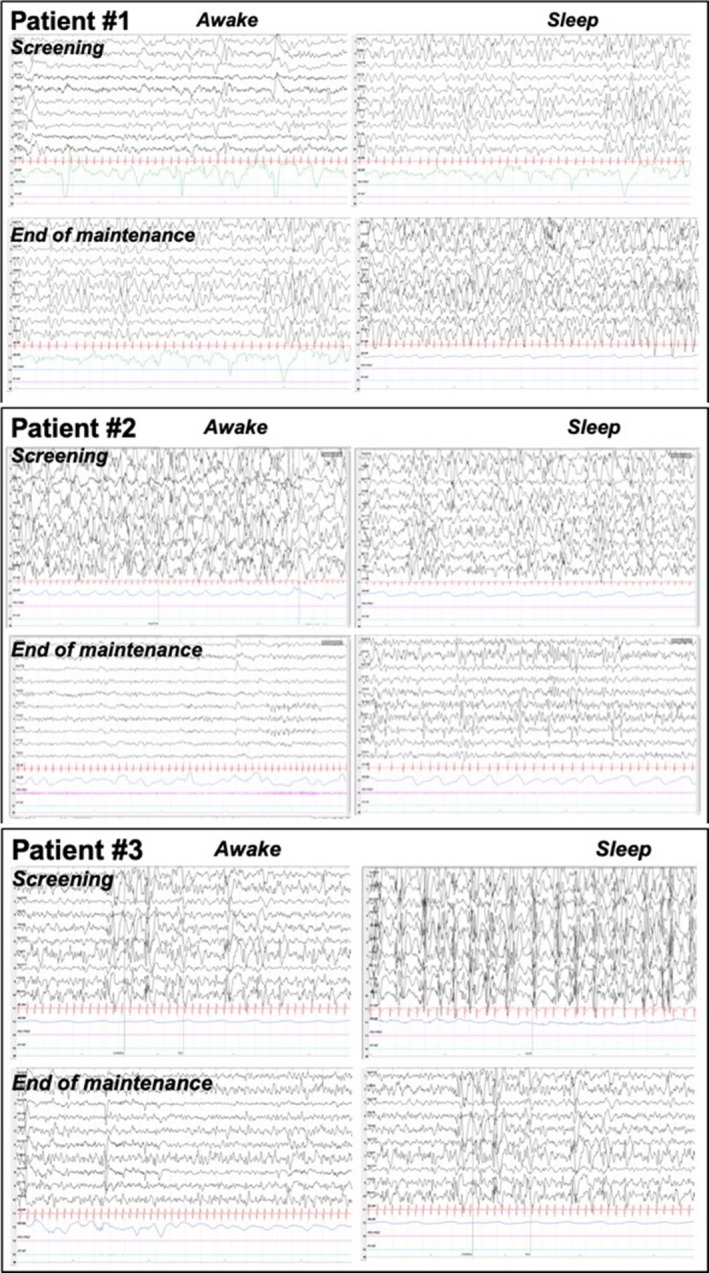
Interictal EEG recordings of the patients at baseline and at the end of maintenance period.

Patient #1 became seizure free with the medium dose, and remained seizure free after the discontinuation of radiprodil up to and including the final study visit at 18 months. Patient #2 and #3 had a clinical improvement with a decrease of epileptic spasms from several daily clusters of spasms lasting for several minutes to isolated epileptic spasms one to three times a day with the high dose of radiprodil. This improvement remained stable for the two patients over the maintenance period. Both patients experienced recurrence of the clusters of spasms when radiprodil was withdrawn. The interictal EEG recordings for each patient were similar at baseline and at the end of the maintenance period (Fig. 4).

All three study participants experienced a total of 20 Treatment Emergent Adverse Events (TEAEs) during the study. The most common nonserious TEAEs (occurring in >1 study participant) were vomiting (*n* = 2 events in two participants) and pyrexia (*n* = 4 events in two participants). No study participants discontinued the study due to TEAEs or experienced any drug‐related TEAEs.

The study was terminated due to insufficient enrollment. To date, after 10–11 months after the treatment with radiprodil, the study participant #1 remains seizure free. After an initial relapse of spasms following radiprodil discontinuation, the two other infants eventually became spasm‐free after few months with a combination of AEDs and the use of the ketogenic diet (Table 2).

## Discussion

Radiprodil is an orally active, negative allosteric modulator of the NR2B subunit of the NMDA receptors. We report here the preclinical and first clinical data in infants obtained with this compound. Radiprodil exerted anticonvulsant efficacy with a high potency in an animal model of generalized clonic seizures. Interestingly, radiprodil displayed much stronger anticonvulsant effect in young rat pups than in adult animals.

The age‐dependent anticonvulsant effect of radiprodil was consistent with data obtained with another NR2B‐selective compound, ifenprodil.^22^, ^23^ The higher efficacy of radiprodil at that specific developmental age is likely to be explained by the developmental changes in expression of NR2B‐containing NMDA receptors, which predominate in the developing brain and are progressively outnumbered by NR2A‐containing NMDA receptors during adulthood. This developmental pattern of NR2B expression of the NMDA receptors is conserved across species, including humans.^30^, ^31^, ^32^ This lead to hypothesize a specific and stronger seizure protective effect of radiprodil in infants.

Infantile spasm syndrome is the most frequent infantile epileptic syndrome despite its low incidence. There is no wide consensus for the treatment of ISS, although SoC is based on hormonal therapy (ACTH or prednisolone) and vigabatrin. Although steroids and vigabatrin used as monotherapy or in combination show good level of efficacy for the majority of patients, ISS remains a therapeutic challenge associated with a significant patient burden that include high level of intellectual disability and high risk of developing other types of pharmacoresistant seizures. There are limited data providing evidence for the selection of the third or later lines of treatments. Accumulating prospective and retrospective studies suggest the ketogenic diet is beneficial, while conventional antiepileptic drugs do not provide significant seizure reduction.^33^ Felbamate is an antiepileptic drug that interferes with NMDA receptors by blocking the NR1A and NR2B subunits and also acts by blockage of the voltage‐gated sodium channels, potentiation of GABA receptor currents, and inhibition of voltage‐gated calcium currents.^34^, ^35^ In our centre, we recently reported that 28% of patients became spasm‐free with felbamate at a mean daily dose of 34.6 mg/kg after failure of at least three treatments (vigabatrin, steroids and also for most of them the ketogenic diet).^29^ Until recently very few ISS patients have been reported as successfully treated by felbamate (*n* = 15).^36^, ^37^ A 72% median reduction of spasms on felbamate compared to baseline was reported in 11 patients using 2‐h video‐EEG recordings as a primary endpoint.^37^ These clinical data contribute to the hypothesis that modulation of NMDA receptors, and radiprodil can have a therapeutic role for ISS.

The phase 1b clinical study (NCT02829827) reported here, involved several centers in Europe. The study, however, was terminated because of the challenge in recruiting a sufficient number of patients within a reasonable time frame. Among the three infants enrolled to the study, one patient became spasm‐free, and two patients had clinical improvement followed by a worsening after radiprodil discontinuation. This first clinical use of radiprodil in infants indicates the compound was generally well tolerated. There were no treatment related serious adverse events, no sedation, and no behavioral changes observed. In all three patients, the pharmacokinetic data were closely consistent with the predicted exposures from the PBPK modeling.

A number of factors contributed to the difficulty in recruiting infants into the study. For safety purposes, the design of the study required that the first two infants treated by radiprodil remained admitted to the in‐patient department for about 2 months. This was a limitation for most of the centres and an extra burden for families. In addition to the low incidence of the syndrome, the more recent use of a combination of vigabatrin and oral steroids therapy has, most likely, further decreased the number of infants meeting the selection criteria. Indeed, 72% of the infants are spasm‐free after treatment with ACTH and vigabatrin combination.^15^

Targeting NR2B subunit of the NMDA receptors appears to be a promising therapeutic strategy based on the receptor neurobiology coinciding with the onset of seizures, its predominant expression in infancy, the initial experimental evidence of radiprodil reported in the present study and our clinical experience with felbamate in ISS. The favorable safety, tolerability, and pharmacokinetic profile of radiprodil, its potential efficacy and the high unmet need warrant further studies in infants suffering from resistant seizures.

## Conflict of Interest

Stéphane Auvin has served as consultant or received honoraria for lectures from Advicenne Pharma, Biocodex, Eisai, GW Pharma, Novartis, Nutricia, Shire, UCB Pharma, Ultragenyx, Zogenyx. He has been investigator for clinical trials for Advicenne Pharma, Eisai, UCB Pharma and Zogenyx.

Blandine Dozières‐Puyravel has received honoraria for lectures from Eisai and UCB Pharma. She has been co‐investigator for clinical trials for Advicenne Pharma, Eisai, UCB Pharma and Zogenyx.

UCB biopharma was the clinical study sponsor. DS, JC, AA, RK, and PM are or were at the time this study was conducted full time employees of UCB Pharma, a pharmaceutical company active in the drug discovery, and development of new anticonvulsants.
